# The role of CDC25C in cell cycle regulation and clinical cancer therapy: a systematic review

**DOI:** 10.1186/s12935-020-01304-w

**Published:** 2020-06-03

**Authors:** Kai Liu, Minying Zheng, Rui Lu, Jiaxing Du, Qi Zhao, Zugui Li, Yuwei Li, Shiwu Zhang

**Affiliations:** 1 Department of Pathology, Tianjin Union Medical Center, Tianjin, 300121 People’s Republic of China; 2 Department of Pathology, Tianjin Nankai Hospital, Tianjin, People’s Republic of China; 3 Departments of Colorectal Surgery, Tianjin Union Medical Center, Tianjin, 300121 People’s Republic of China

**Keywords:** CDC25C, Cell cycle, G2/M progression, DNA damage, Cancer

## Abstract

One of the most prominent features of tumor cells is uncontrolled cell proliferation caused by an abnormal cell cycle, and the abnormal expression of cell cycle-related proteins gives tumor cells their invasive, metastatic, drug-resistance, and anti-apoptotic abilities. Recently, an increasing number of cell cycle-associated proteins have become the candidate biomarkers for early diagnosis of malignant tumors and potential targets for cancer therapies. As an important cell cycle regulatory protein, Cell Division Cycle 25C (CDC25C) participates in regulating G2/M progression and in mediating DNA damage repair. CDC25C is a cyclin of the specific phosphatase family that activates the cyclin B1/CDK1 complex in cells for entering mitosis and regulates G2/M progression and plays an important role in checkpoint protein regulation in case of DNA damage, which can ensure accurate DNA information transmission to the daughter cells. The regulation of CDC25C in the cell cycle is affected by multiple signaling pathways, such as cyclin B1/CDK1, PLK1/Aurora A, ATR/CHK1, ATM/CHK2, CHK2/ERK, Wee1/Myt1, p53/Pin1, and ASK1/JNK-/38. Recently, it has evident that changes in the expression of CDC25C are closely related to tumorigenesis and tumor development and can be used as a potential target for cancer treatment. This review summarizes the role of CDC25C phosphatase in regulating cell cycle. Based on the role of CDC25 family proteins in the development of tumors, it will become a hot target for a new generation of cancer treatments.

## Background

According to the GLOBOCAN 2018 global cancer morbidity and mortality estimates, the number of new cancer cases and deaths was 11.8 million and 9.6 million, respectively. These numbers are growing rapidly, and cancer will be the first and most important issue in every country, and the most important obstacle to increase life expectancy [1]. Therefore, understanding the mechanism of tumorigenesis is greatly helpful in inhibiting tumorigenesis, slowing down the development of tumors, and early diagnosis of tumors. Although each type of cancer can be caused by a variety of factors, the majority are characterized by cell cycle disorders and irregularities, such as deletions, overexpression, or mutations in proteins that control this process.

The dual-specificity phosphatase cell division cycle-25C (CDC25C) plays an important role in the regulation of serine/threonine kinase activity involved in cell cycle. CDC25C can promote mitotic cell G2/M transition by triggering cyclin-dependent kinase-1 (CDK1) dephosphorylation to activate the cyclin B1/CDK1 complex [2]. The downregulation of CDC25C induces cell cycle arrest in G2/M phase in response to DNA damage via p53-mediated signal transduction, and its abnormal expression is associated with cancer initiation, development, metastasis, occurrence and poor prognosis. Many studies have shown that CDC25C is highly expressed in lung [3, 4], liver [5], gastric [6], bladder [7], prostate [8, 9], esophageal [10], and colorectal cancers [11] and in acute myeloid leukemia [12], correlated to poor prognosis and low survival rates. Investigating the underlying mechanisms of CDC25C in cancer initiation and progression may provide a new insight into the diagnosis and treatment of human tumors.

## Structure of CDC25 family

CDC25A, CDC25B, and CDC25C are three subtypes of the CDC25 phosphatase family, the molecular weight between 53 and 65 kDa, and with chain lengths of 524, 580, and 473 amino acids, respectively. The human CDC25 protein family structure has a conserved catalytic domain and a regulatory domain. The latter can be divided into the N-terminal and the C-terminal regulatory domains [13]. In the N-terminal regulatory domain, which has phosphorylation and ubiquitination site that regulate phosphatase activity, there are large differences between the three isoforms: sequence similarity is less than 25% and they vary in length [14]. There are sites that regulate phosphatase activity, stability, and their interaction with other proteins and modifications to these sites involve normal cell cycle control and checkpoint response signals [5]. At the same time, the N-terminal region contains the nuclear localization sequence (NLS) and the nuclear export sequence (NES), which determine the subcellular localization of CDC25 phosphatase [13]. The C-terminal regulatory domain has 60% identity between isoforms, contains a catalytic site for phosphatase, and is capable of dephosphorylating protein substrates as rapidly as full-length proteins [15]. At present, X-ray crystallography has helped us understand the structure of the catalytic domains of CDC25A and CDC25B. These results proposed that the catalytic site of CDC25B can bind to oxygen anions, unlike CDC25A. The C-terminal region also presents significant conformational diversities [16].

CDC25 phosphatase is part of the tyrosine phosphatase family and its core region contains the characteristic motif HCX5R and a catalytic aspartate residue. In C-(X)5-R, H represents the highly conserved histidine residue, C represents a catalytic cysteine, and X5 is a ring composed of 5 residues. All of the amide hydrogens are associated with the phosphate of the substrate. R is arginine, which is needful for phosphorylating amino acid binding with matrices [17, 18]. The overall folding of CDC25 is different from that of other tyrosine phosphatases; however, the active site loop can be superimposed on the active sites of a variety of other tyrosine phosphatases. A key feature of CDC25 is that its active site is flat, with no distinct grooves for binding proteins or small molecule substrates, which is consistent with lack of activity or specificity for peptide substrates [15], suggesting a broad interaction.

## Cell cycle regulation of CDC25A and CDC25B

CDC25A is mainly localized in the nucleus, and controls G1/S progression by dephosphorylation-dependent inactivation of Cyclin E/CDK2 and Cyclin D/CDK4-6; G2 phase progression by dephosphorylation of CDK1 and activation of cyclin B1/CDK1 complex is a limiting step [19]. CDC25B is considered to be the “initiator” phosphatase and is located in the nucleus during interphase, but shuttles back and forth between the cytoplasm and nucleus during entire cell cycle. It translocate to the cytoplasm during the G2 phase and activates the cyclin B1/CDK1 complex [16], and then re-enters the nucleus to initiate mitosis [20].

## Phosphatase activity and subcellular location of CDC25C

The phosphorylation activity of CDC25C during interphase is low and localize in the cytoplasm. The cytoplasmic localization depends on CDC25C amino acid sequence 201–258, which contains specific binding sites with 14-3-3 protein. The combination of 14-3-3 and CDC25C can make CDC25C location in the cytoplasm [16]. Therefore, CDC25C is regulated by 14-3-3 protein by means of chelating in the cytoplasm [16]. It has also been reported that phosphorylation of CDC25C on Ser216 and Ser287 at the interphase prevents its activation and promotes cytoplasmic location by binding to 14-3-3 protein [21].

During interphase, CDC25C shuttles from the cytoplasm to the nucleus. The localization of CDC25C during the cell cycle is affected, to a certain extent, by alterations in the rate of import and export [20]. Boutros et al. confirmed that NES sequence in CDC25C regulated the location of cytoplasm and nucleus. When Ser191 and Ser198 in NES were phosphorylated, it could facilitate nuclear translocation of CDC25C [22].

## Physiological function of CDC25C

CDC25C is a representative bispecific phosphatase which hydrolyzes Tyr and Ser/Thr phosphate [13]. Although CDC25A and CDC25B activate the cell cycle cyclin B1/CDK1 complex, CDC25C is in charge of promoting and maintaining complete cyclin B1/CDK1 activation, and ultimately determines the G2 checkpoint [23]. CDC25C acts on the CDK1 Thr14 and Tyr15 residues, dephosphorylating them, and activating this complex into mitosis [16]. Dephosphorylation of CDK1 regulated by CDC25C and nuclear entry of the cyclin B1/CDK1 complex are key mechanisms regulating the initiation of cell division. When CDC25C activity is inhibited, the activity of the cyclin B1/CDK1 complex will also be inhibited, therefore CDC25C plays a crucial part in controlling the cell cycle [24].

In G2/M progression, activation of CDC25C requires dissociation from 14-3-3 protein and phosphorylation in various sites within the N-terminal domain, such as Thr48, Thr67, Ser122, Thr130, and Ser214 [16]. *p*53, and checkpoint protein kinases CHK1 and CHK2 regulate phosphorylation of CDC25C and inhibit the activity of CDC25C when DNA damage occurs, which results in CDC25C breakdown in the cytoplasm [25]. This will prevent the activation of the cyclin B1/CDK1 complex, arresting the cell cycle in the G2/M phase, and the mutated region will not be transmitted to the progeny cells. In addition, CDC25C can be inactivated by Wee1 and Myt1 kinase [26].

## Reciprocal activation between cyclin B1/CDK1 and CDC25C

Cyclin B1/CDK1 is the first kinase to phosphorylate and activate CDC25C in vitro. Cyclin B1 is a regulatory protein involved in mitosis, and it forms a complex with CDK1 to form the M phase promoting factor (MPF). We currently know that the CDK family contains a total of 14 serine/threonine protein kinases that coordinate cell cycle progression and are responsible for entry into mitosis [27]. Activity of numerous cell cycle-associated proteins are modulated by its subcellular localization; for example, the initiation of mitosis requires activation of MPF, the cyclin B1/CDK1 complex, which localizes in cytoplasm during interphase and translocate to the nucleus after the beginning of mitosis, followed by rupture of the nuclear membrane. Nuclear export sequence in cyclin B1 mediates the nuclear export of MPF. When the regulatory sequence is impaired, cyclin B1 will accumulate in the interphase nucleus. Thus, MPF alternates between the nucleus and cytosol during interphase [28]. Cyclin B1 cytoplasm localization is regulated by an amino terminal region called the cytoplasmic retention sequence (CRS), which is 42 residues long. If this region is inhibited, cyclin B1 will be localized in the nuclear throughout the mitotic cycle [29]. Cyclin B1 is promoted to enter the nucleus before mitosis when the NES of cyclin B1 is inactivated. DNA damage can deter the inactivation of the NES, thereby force cyclin B1 to stay in the cytoplasm [16]. Upon entry into the cytosol, a positive feedback loop among a large number of endogenous cyclin B1/CDK1 complexes and CDC25 phosphatases is activated, inducing the accelerated entry of the cell into mitosis. Therefore, the significance of the cyclin B1/CDK1 complex in harmonizing the cell into mitosis can be confirmed by the above effects [28]. However, cyclin B1/CDK1 complex activity is the result of co-regulation of CDK1 phosphorylation, cyclin B1 accumulation, and intracellular localization of cyclin B1 [30].

It has been reported in the literature that cell division begins after the cyclin B1/CDK1 complex enters the nucleus; at the same time, the complex activation and turn into the nuclear requires CDC25C catalysis. During the interphase, CDK1 is phosphorylated to render the cyclin B1/CDK1 complex in an inactive state. Pre-dividing CDC25C activates the cyclin B1/CDK1 complex, which in turn phosphorylates CDC25C, and further activates cyclin B1/CDK1 complex binding, both of which are compulsory steps of the autocatalytic activation loop. First, CDC25C dephosphorylates Thr14 and Tyr15 in the CDK1 to activate it, promoting mitotic entry and antagonizing Wee1 function [22]. Activated cyclin B1/CDK1 phosphorylates CDC25C at Thr48, Thr67, Thr138, Ser205, and Ser285 to activate it. It has been reported that phosphorylation of Ser285 may be a key point for positive feedback activation of CDC25C by cyclin B1/CDK1. On one hand, since phosphorylation at this site helps to maintain mitosis by blocking CHK1-mediated phosphorylation of Ser287, it can promote the recruitment of serine/threonine protein phosphatase-1 (PP1) to CDC25C and enhance the combination of them [21]. Before the onset of cell division, CDK1 is activated by dephosphorylation of CDC25, which helps the cyclin B1/CDK1 complex to rapidly transfer from the cytoplasm to the nucleus before the start of cell division, regulating the initiation of cell division [31]. Dephosphorylation of CDK and entry of the cyclin B1/CDK1 complex into the nucleus are key mechanisms regulating the initiation of cell division [32].

de Gooijer et al. proposed a node-based model of G2 checkpoint regulation. The action of the central Cyclin B1/CDK1 node is regulated by the concerted but opposing activities of the Wee1 and CDC25C [14]. There is a positive feedback loop between CDK1 and CDC25C. CDK1 in the cytoplasm can activate CDC25C by phosphorylating the Thr48/Thr67 site before CDC25C entries into the nucleus, which makes CDC25C obtain the targeting activity of CDK1. The nuclear location of CDC25C also inhibits CHK1 nuclear phosphorylation, and inhibition of CHK1 nuclear phosphorylation reversely regulate CDC25C nuclear decision state (NDS) [14]. Wee1 regulating nuclear CDK1 phosphorylation in the Tyr15 site can result in Cyclin B1/CDK1 complex inactivation, cell cycle arrest in G2 and DNA damage repair [28]. Continuously activated CDC25C further dephosphorylates CDK1, which can break the balance of between CDC25C and Wee1 [11]. Down expression of cyclin B1 can inhibit cell proliferation and arrest of G2/M phase [26, 33].

## CDC25C responds to DNA damage as a checkpoint regulatory protein

Cell division is a highly regulated event, characterized by complete and accurate transfer of genomic information to daughter cells. Genomic instability is a common feature of cancer cells, promoting the accumulation of oncogenic mutations, which are key factors in the induction of cancer and a major obstacle to cancer treatment. DNA damage response mechanisms are believed to prevent or delay genetic instability and tumorigenesis, acting as a barrier against cancer. The response of cells to DNA damage is primarily coordinated by two distinct kinase signaling cascades, the Ataxia telangiectasia-mutated gene (ATM) and Rad3-related serine/threonine kinase (ATR)/checkpoint kinase 1 (CHK1) and the Ataxia telangiectasia mutated serine/threonine kinase (ATM)/checkpoint kinase 2 (CHK2) pathways, named cell cycle checkpoint proteins [34].

Cell cycle checkpoints are central mechanisms in eukaryotic cells that control DNA replication and repair, mitosis, and cytokinesis, and couple the completion of these events with the occurrence of mitosis. However, harmful stress promotes activation of checkpoint proteins, arresting cell cycle in time; if checkpoint activation does not initiate, the cell will enter apoptosis [24]. Associated with cell cycle checkpoint initiation, the CDC25 phosphatase family are the primary candidates for the CHK1 and CHK2 checkpoint kinases, mainly in the S and G2/M phase, DNA damage-induced phosphorylation, thereby activation, of these proteins. The key task of CHK1 and CHK2 is to signal the proximal checkpoint kinase from the phosphatidylinositol 3-kinase family [35]. Activation of CHKs inactivates CDC25C in a phosphorylation and ubiquitination-dependent manner through Ser216, leading to its degradation, and thereby preventing the activation of downstream signaling pathways such as p21 and cell cycle B1, which is required for entry into G2/M phase [36]. In addition, phosphorylation of CDC25C on Ser345 or Ser317 by ATM, ATR, or CHK1 results in the arrest of S or G2/M phase. The activation of CHK2 is carried out through an ATM-dependent method by phosphorylation of Thr68. It has also been reported that the G2/M checkpoint produces a 14-3-3 binding site by phosphorylation of CDC25C at Ser287, inhibiting M phase entry [37]. Checkpoint dysfunction is considered to be a pathological marker of tumor transformation and progression [10]; therefore, it plays an important role in inhibiting tumor development, as a pathway necessary to maintain genomic stability [14, 35].

## CHK1 negative regulation of CDC25C in response to DNA damage

The ATR/CHK1 pathway is usually up-regulated in tumors and is believed to promote tumor growth. ATR is mainly involved in G1 phase cell cycle arrest, and comes into play when dissociated single-strand DNA breaks [38]. Phosphorylation of ATR at Ser317 and Ser345 promotes CHK1 function and induces its autophosphorylation at Ser296 [39]. CHK1 phosphorylates and inhibits CDC25C, and this is the major effector for mitotic arrest. Specifically, CHK1 exerts its phosphorylation function at the CDC25C Ser287 residue, and induces CDC25C degradation [40]. Subsequently, 14-3-3 associates with CDC25C, resulting in phosphorylation of Thr138, causing dissociation from CDC25C [41]. Both ATM and ATR phosphorylation events occur in the nucleus and trigger phosphorylation of the Ser317 and Ser345 residues of the downstream target CHK1 [42, 43], induce autophosphorylation of Ser296 residue on CHK1, and enable CHK1 to achieve its induced G2 arrest [44]. These phosphorylation events are necessary to pass the checkpoint signal to the downstream effector.

In interphase, since the center of action of CHK1 is the nucleus, CDC25 phosphatase in the cytoplasm is inhibited. At the same time, CDK1 cannot be activated to complete nuclear translocation. Once the CDK1 activation process is initiated (possibly in G2 phase), the Ser286 and Ser301 sites of CDK1 will be auto-phosphorylated. This phosphorylation induces the translocation of CHK1 from the nucleus to the cytoplasm, and interference with this process leads to mitotic delay [45]. The nuclear-cytoplasm cycle of CHK1 means that inhibition of nuclear localization of CHK1 leads to cytoplasmic accumulation and prevents CHK1 degradation from increasing its cellular levels [44]. CDK1-induced phosphorylation of CHK1 inhibits its function in the nucleus, which results in the activation of positive feedback loop. In addition, CHK1 antagonizes serine/threonine-protein kinase 1 (PLK1) function and promotes nuclear translocation by phosphorylating the Ser642 site of Wee1 [46].

Wee1 and CDC25C are two ideal candidates for CHK1 regulation of CDK1, achieved mainly by adjusting the balance between their subcellular localization. The target of CHK1 in Wee1 regulation is the phosphorylation site Ser642 site, as well as CDK1 phosphorylation on Tyr15, which inhibits the kinase activity of CDK1 [35]. In contrast, CHK1 phosphorylates CDC25C on Ser216, indicating that it can undergo extranuclear translocation and prevent CDK1 phosphatase activity. At the same time, a 14-3-3 protein binding site is created, causing nuclear export of CDC25C, preventing activation of the cyclin B1/CDK1 complex. Therefore, activation of CHK1 prevents premature cell entry into mitosis [47, 48].

Overexpression or decreased expression of CHK1 also occurs in certain types of tumors, including breast, ovarian, cervical, and neuroblastoma [49, 50]. It is worth noting that its expression is often positively correlated with tumor grade and disease recurrence [47].

## CHK2 phosphorylates CDC25C promoting cell cycle arrest

Mutations and deletions of ATM/CHK2 are often found in tumors and lead to their development [51]. The pathway presented here is the barrier to prevent tumor growth and cancer. ATM is one of the most sensitive effectors of cell cycle changes aroused by DNA damage and belongs to the phosphatidylinositol kinase-associated family [52]. ATM functions in early stage of cell signal transduction, and ATM deficient cells show abnormal cell cycle and tumor susceptibility. Double-chain breakage of DNA marks that beginning of ATM activation, mainly partake in G2 checkpoint stagnation [53]. CHK2 is a protein that is stably expressed during cell cycle and appears to be inactive without DNA damage in the form of inactive monomers in nucleus. The activation of CHK2 involves dimerization and autophosphorylation at residues Thr 383, Thr 387, Ser 546, and Ser 516. Compared with ATR-CHK1, ATM-CHK2 is dedicated to providing cells with a rapid protective response to the lethal effects of DNA damage.

ATM activates CHK2, resulting in phosphorylation of CDC25C, rendering it unable to activate the cyclin B1/CDK1 complex. CHK2 phosphorylates CDC25C Ser216 and Ser287 residues, thereby producing a binding site for 14-3-3, which in turn promotes CDC25C translocation to the cytoplasm,terminating its contribution in the cell cycle. Additionally, CHK2 phosphorylates the N-terminal activation domain of P53 to regulate the response of P53 to DNA damage. CHK2 also phosphorylates P53 Thr18 and Ser20 to promote p21 accumulation and to maintain G2/M arrest. Activation of ATM-CHK2 also potentiates cyclin B1/CDK1 block mediated by ATR-CHK1 [54]. Previous studies on curcumin (CUR) tumor suppression function show that inhibition of ATM production completely reverses CUR-induced cell cycle arrest and anti-angiogenesis. Furthermore, CUR exerts its antitumor activity by targeting the ATM-CHK2-P53 signaling pathway [55].

Mutation or down-regulation of CHK2 has been found in human primary breast [56, 57], colon [58], lung [59], vulvar [60], bladder [61], and ovarian cancers [62], and in osteosarcoma [63] and lymphoma [64]. Phosphorylation of the CHK2 Thr68 site is particularly evident in p53 mutant cell lines.

## Wee1 antagonizes CDC25C in G2 checkpoint regulation

Two groups of mutations have been reported to alter the regulation of mitosis, the first type of mutation causes a delay in cell cycle and G2 arrest, and the other type of mutation is characterized by a ‘Wee’ phenotype due to accelerated cell entry into mitosis. CDK1 and CDC25 belong to the first class, which is required for encoding normal proteins for mitosis; the second is a dominant activating mutation of CDK1, and WEE1 belongs to this class. WEE1 and CDC25 are dose-dependent mitotic inhibitors and activators, respectively [65].

Wee1 is a G2 checkpoint regulator, which prevents entry into mitosis in the presence of DNA damage [66]. There are two mechanisms by which G2 checkpoints can be initiated as response to DNA damage: CHK1 phosphorylation of CDC25C and CDK1, and Wee1 kinase phosphorylation [26]. Since the G2 checkpoint responds to DNA damage, CDK1 phosphorylates Wee1 and CDC25C when DNA damage occurs, resulting in activation of Wee1 kinase and inactivation of CDC25C phosphatase [67]. Next, activation of CDK1 is negatively regulated by Wee1, by phosphorylating its Thr14 and Tyr15 sites [68], enabling cyclin B1/CDK1 complex inactivation, which results in cell cycle arrest in G2 phase, leads to a stage of DNA damage repair, suspending mitosis.

Wee1 is differentially located during the cell cycle. It is ususally localized in the nucleus to regulate MPF deactivation, until CDC25C inhibits its effect. Phosphorylation of endogenous CDC25C Ser 287 is usually high during the interphase; meanwhile, Wee1 Ser549 phosphorylation is low. After DNA damage and checkpoint initiation, activity of Wee1 is increased [69, 70]. Degradation of Wee1 can be achieved by PLK1 phosphorylation of Ser53 [71].

G2 checkpoint proteins are a fulcrum for regulating G2/M conversion, and also a bridge between DNA state and cell cycle progression. By regulating the expression of cyclin B1/CDK1, PLK1, CDC25C, CHK1–CHK2, and Wee1 the cell cycle is ensured, cells correctly divide and the precise genetic material is passed onto the daughter cells. In the event of DNA damage, timely initiation of the repair program induces G2/M phase arrest, providing an opportunity for damage repair and preventing erroneous DNA from being transmitted. Based on the unique cell cycle regulation function of G2 proteins, future research will provide us with new understandings about cell cycle and DNA information transmission.

## P53-mediates DNA damage repair and cell cycle arrest

*p*53 is a gene that has been found to be highly associated with human tumors, being mutated in more than half of the malignant tumors, suggesting that genetic alterations in this gene may be the primary cause of human tumorigenesis. The effect of P53 on protein expression appears to be significant in the cell cycle arrest response caused by DNA damage [72]. It is highly induced by a variety of stress signals, such as DNA damage, hypoxia, activation of oncogenes, and nutrition deficiencies, which lead to cell cycle disorders, apoptosis, aging, differentiation, and anti-angiogenesis [73]. When exposed to these stimuli, P53 exerts its functions to avoid tumorigenesis. The main regulatory pathways that P53 activates are: initiation of apoptosis, repair of DNA damage, and cell cycle arrest [70]. The main regulatory function of P53 in the cell cycle is the monitoring and transcriptional activation of G1 and G2/M phase correction points [74]. P53 mediates *p*53-dependent cell cycle arrest, depending on activation of Tyr15 and Thr14 phosphatase. P53 can initiate a phosphorylation cascade in case of DNA damage in order to avoid cell cycle arrest before M phase, trough the inhibition of cyclin B1/CDK1, necessary for M phase conversion [75]. The downstream effector of P53, the multiprotein p21 or CIP1, can independently arrest G2,but this only works when DNA damage occurs, and this cycle of stagnation cannot be maintained for a long without P53 involvement [72].

CDC25C is a novel partner for down-regulation of P53 transcription. The CDC25C gene contains a P53 binding site and P53 inhibition by the CDC25C promoter induces G2/M arrest after DNA damage. There are two mechanisms inhibition of CDC25C by P53: first, the p53 response element inhibits the CDC25C promoter by the CDE/CHR element, and p21 also mediates DNA damage-induced CDC25C gene down-regulation by this element. The second one is related to the synergistic effect of Sp1-like cytokines and P53 [76, 77].

P53-mediated cell cycle arrest is mainly caused by transcriptional activation of p21/WAF1 [78]. As the first transcriptional target of P53, p21 acts a mediator of P53 and plays a vital role in the regulation of cell cycle progression and stagnation [79]. The p21-encoded protein is an inhibitor of cyclin-dependent protein kinase that binds to the Cyclin E/CDK2 and Cyclin D/CDK4 complexes, leading to cell cycle G1 arrest [80]. It also promotes CDK1 Tyr15 phosphorylation, inhibits cyclin B1/CDK1 activity, and regulates mitotic cell cycle progression [81]. At the same time, three important downstream effectors of cyclin B1, GADD45, and 14-3-3σ, are also involved in G2/M phase arrest. P53 blocks G2/M conversion by decreasing cyclin B1 expression levels and attenuating the activity of the cyclin B1 promoter [82]; GADD45 acts by inhibiting the activity of the cyclin B1/CDK1 complex [83]; 14-3-3σ binds to CDC25C to interfere with the transcriptional regulation of the cyclin B1/CDK1 complex, causing its cytoplasmic translocation [84]. In summary, when DNA damaged arises, P53 regulates cell cycle arrest and prevents DNA replication, giving extra time for thorough DNA damage repair. If it fails, P53 induces apoptosis. If both regulatory pathways of p53 are mutated, cell proliferation will be out of control, leading to cell cancer.

## PLK1 phosphorylates CDC25C at multiple sites to increase its activity

Serine/threonine kinase family member PLK1 plays an important role in regulating the activity of human mitosis [85]. Activation of PLK1 requires phosphorylation of a conserved threonine in the T-loop of the kinase domain, as well as synergistic phosphorylation of G2 phase Aurora-A and its cofactor Bora [86]. The expression of PLK1 largely depends on cell cycle progression, and its level peaks in G2 and M phases. The expression level of PLK1 is positively correlated with its effect, the expression level of PLK1 is also increased in cells with high mitotic rate [87]. The subcellular localization of PLK1 during different stages of mitosis determines its function [88]. PLK1 carries a nuclear translocation signal, allowing its subcellular localization to be strictly regulated in the cell cycle [89]. Recent studies have shown that, although the cytoplasm is the first place where PLK1 is activated, its activity was first determined in the nucleus early in the G2 phase [90]. PLK1 also acts as a mitotic switch to mediate post-injury repair, reducing genetic mutations caused by DNA damage. PLK1 overexpression has been detected in a multiple human tumors, and its expression degree is related with increased proliferation of cancer cells in patients and with poor prognosis [19, 91]. PLK1 controls the development of cancer through a variety of mechanisms, including mitosis, cytokinesis, DNA replication, and cell survival. PLK1 has also been shown to be associated with tumor epithelial-mesenchymal transition and tumor invasion [87].

The signals of nucleus stabilization or translocation mediated by PLK1 phosphorylation can affect CDC25C and Wee1 nucleus accumulation, which can be attributed to the role of PLK1 in the cytoplasm or nucleus [92]. CDC25C phosphorylated by PLK1 in various, increasing its degree of activation, promotes its nuclear translocation. When CDC25C is activated, the inactive cyclin B1/CDK1 complex is dephosphorylated, further increasing its activity to promote G2/M progression and translocate the complex into the nucleus [16]. Researchers found that PLK1 phosphorylates Ser147 and Ser133 of the cyclin B1/CDK1 complex to promote its access to the nucleus [85]. Hydrophobic and acidic residues abound in the NES sequences of cyclin B1 and CDC25C, which may be ideal substrates for PLK1. At the same time, PLK1 phosphorylates the Ser198 residue of the CDC25C NES, causing nuclear translocation of CDC25C [38, 93]. In addition, phosphorylation of PLK1 on Ser53 residues induces the degradation of Wee1 while eliminating the inhibitory effect of Wee1 on CDC25C [34].

Studies have shown that PLK1 and CDC25C are overexpressed in prostate cancer, and PLK1 expression is also associated with high tumor grade. Overexpression of PLK1 can repair mitotic arrest caused by UV by inhibiting the activation of P53 [50]. PLK1 and CDC25C combine with P53 to participate in recovery of mitotic arrest forming a large complex during this process. First, PLK1 activates CDC25C, and this makes dephosphorylates P53 at Ser 15 to inhibit its activity. Together, the three regulate mitotic disorders and provide protection for the correct functioning of the cell cycle [45].

## Aurora A promotes nuclear localization of CDC25C by phosphorylating PLK1

Aurora A, a member of the mitotic serine/threonine kinase family, regulates the progression of mitosis by phosphorylating multiple substrates, directly phosphorylating PLK1 to activate it, and controlling the activation of cyclin B1/CDK1 to promote mitosis; its activity also contributes to mitotic checkpoint response [94]. The promoter of the Aurora A gene contains specific elements (CDE/CHR sequences) for regulating the transcription of the G2 phase of the cell cycle. Aurora A has been shown to be necessary for initiation of mitosis and cytokinesis, spindle maturation, and isolation, and G2 to M progression [95].

During mitosis, Aurora A phosphorylates PLK1 to promote nuclear localization of CDC25C. Bora is an important cofactor for Aurora A to mediate PLK1 activation through phosphorylation. Bora interacts with PLK1 to cause a conformational change to allow Aurora A to enter the T-loop of PLK1 and phosphorylate Thr210 to activate PLK1. Activation and activity of PLK1 is not proportional to the concentration of Aurora A, a small quantity of Aurora A can initiate PLK1 activation [86]. PLK1 then phosphorylates the CDC25C Ser198 residue and initiates CDC25C nuclear translocation [96]. When active, CDC25C phosphorylates CDK1 in Tyr15 and promotes cell G2/M progression. A study found that in renal cell carcinoma, the down-regulation of Aurora A kinase induces a decrease in the expression of cyclin B1/CDK1 complex, and inhibits cell proliferation and metastasis by blocking ERK activity, thereby exerting anti-tumor effects [97].

Overexpression or gene amplification of Aurora kinase has been found in a variety of human malignancies, including breast, colorectal, bladder, pancreatic, gastric, ovarian, and esophageal cancers,indicating its role as an oncogene in tumorigenesis [98]. Its mechanism of action is to evade cell cycle checkpoints, promote cell proliferation, and inhibit cell apoptosis. Highly expressed Aurora A is associated with poor patient survival, suggesting that it plays an important role as a cancer therapeutic target [99].

ARID1A is a tumor suppressor, and its mutated form has been confirmed in a variety of cancers, such as endometrial cancer, and is associated with tumor progression and metastasis. ARID1A and Aurora kinase A have synergetic lethal interactions. ARID1A competitively binds to the Aurora kinase A promoter and negatively regulates its transcriptional activity; cells with ARID1A deficiency have increased transcription level of Aurora A, resulting in sustained activation of CDC25C. The Aurora A-CDC25C axis represents a novel strategy for the treatment of colorectal cancer with ARID1A loss of function mutation [100].

## PP1 dissociates and activates CDC25C from the cytoplasm

PP1, which is expressed in almost all eukaryotic cells, catalyzes most of the silk/threonine dephosphorylations [101]. Preliminary studies indicate that PP1 dephosphorylates CDC25C to activate it. Based on a complete study using CDC25C, it was confirmed that the CDC25C N-terminal domain contains a PP1 binding motif, allowing PP1 to directly recognize CDC25C and interact with it [102]. During interphase or after DNA damage, CDC25C is phosphorylated in Ser287 (*Xenopus laevis*, human Ser216), which inhibits the ability of CDC25C to promote M phase switching; then, binds to 14-3-3 protein to isolate CDC25C in the cytoplasm. PP1 is primarily responsible for the dephosphorylation of CDC25 S287, thereby releasing CDC25 isolation upon mitotic entry. Dephosphorylation of Ser287 is mediated by the direct action of PP1 and its docking site on the N-terminal domain of CDC25C [21]. Upon entry into the mitotic phase, CDK2 catalyzes Thr138 phosphorylation of CDC25C, which dissociates CDC25C from 14-3-3 and restores CDC25C activity by PP1-mediated Ser287 dephosphorylation. The study also found that 14-3-3 binds to Ser287 to protect CDC25 from premature dephosphorylation [102].

Phosphorylation at Ser285 is key for positive feedback activation of CDC25C by cyclin B1/CDK1 complex, and CDK1 may be the only kinase among the mitotic proteins that directs Ser285 phosphorylation [103]. Phosphorylation of Ser285 by CDK1 greatly increases the recruitment of PP1 to CDC25C, enhancing the interaction between the two, thereby accelerating the phosphorylation and mitotic entry of Ser287. Upon entry into the M phase, the inhibition of CDC25C was first initiated by dissociation of 14-3-3 and then by PP1-mediated Ser287. Phosphorylation of Ser285 on CDC25C facilitates maintaining the mitotic state by preventing CHK1-mediated phosphorylation of Ser287 [104].

## Pin1 catalyzes conformational change of CDC25C and promotes its dephosphorylation

The prolyl isomerase Pin1 is a P53 binding protein [105]. It participates in the cell cycle progression through regulation of cell cycle-related proteins such as CDK1, CDC25C, Wee1, Myt1, and PLK1, and regulates G2/M progression. Pin1 is a peptidyl-prolyl cis/trans isomerase, which specifically recognizes phosphorylated serine or threonine and proline, originally identified in a yeast two-hybrid screen [106]. Pin1 is able to change the function of the protein it is interacting with [107]. Pin1 participates in the regulation of cell cycle progression, apoptosis, control of key protein expression, proliferation, and oncogenic transformation in a phosphorylation-dependent manner [108, 109]. Pin1 plays an important catalytic role in many cellular events by affecting the conformational changes of the pSer/Thr-Pro motif [110]. Overexpression of Pin1 promotes tumor growth, while its inhibition leads to tumor cell apoptosis [111, 112]. Pin1 plays an important role in tumorigenesis, and it has been found to be overexpressed in many cancers, including breast cancer [113], hepatocellular carcinoma [114], non-small cell lung cancer [115], esophageal squamous cell carcinoma [116], prostate cancer [117], oral squamous cell carcinoma [118], as an effective anticancer target [119].

Studies have found that Pin1 plays an important role in regulating CDC25C phosphatase activity. Pin1 was originally thought to dephosphorylate CDC25C, negatively regulating its function and inhibiting the activation of the cyclin B1/CDK1 complex; however, subsequent studies have shown that Pin1 can also activate CDC25C. Since Pin1 contains a pSer/pThr binding site, a protein with a this motif is preferentially identified as an isomerization substrate. The five pSer/pThr-Pro sites in the mitotic form of *X. laevis* CDC25C are potential Pin1 substrates. Pin1 catalyzes the conformational change of CDC25C and promotes its dephosphorylation by phosphoprotein phosphatase 2A (PP2A), which is considered to be a conformation-specific phosphatase. Pin1 promotes the removal of CDC25C phosphate by PP2A1 by catalyzing the isomerization of specific pSer/Thr-Pro motifs. Pin1 stably binds to CDC25C through pThr48-Pro and pThr67-Pro sites, promoting mitosis and microtubule assembly [120, 121].

It is known that Wee1 protein kinase negatively regulates CDK1 activity by phosphorylating the Thr14 and Tyr15 residues of CDK1, while Pin1 binding to Wee1 can neutralize the inhibitory effect of Wee1 on CDK1 [122], enhance the activity of cyclin B1/CDK1 complex, regulate G2/M progression, and promote Pin1 in cell cycle control and cancer.

## CDC25C dephosphorylates ASK1 to inhibit its activity during the interphase

Apoptosis signal-regulated kinase 1 (ASK1), also known as mitogen-activated protein kinase kinase kinase 5 (MAP3K5), plays a major role in response to various stress stimuli, and in cell signal transduction and biological function regulation [123]. Under normal conditions, ASK1 is often inactive; however, under pathological conditions, ASK1 is activated by the dissociation and oxidation of thioredoxin (TRX), and its activity is often associated with apoptosis levels [124]. ASK1 activation is regulated by multiple steps, including oligomerization, phosphorylation, and protein–protein interactions [125]. ASK1 acts as an upstream regulator for the activation of p38MAPK and c-Jun N-terminal kinase (JNK) regulatory factors. Importantly, ASK1 is only activated under pathological conditions, providing a new target that can disrupt the pathological rather than steady-state function of downstream p38MAPK and JNK signaling pathways [124].

At the interval CDC25C inhibits ASK1, dephosphorylating pThr838 in its activation loop. Overexpression of CDC25C inhibits ASK1-mediated apoptosis. By inhibiting the expression level of CDC25C, the activity of the interphase ASK1 and its downstream targets can be increased, indicating that CDC25C negatively regulates ASK1 activity. During mitotic arrest, CDC25C phosphatase activity was enhanced, but the affinity for ASK1 was significantly reduced, indicating a decrease in the binding of hyperphosphorylated CDC25C to ASK1. These findings indicate that CDC25C negatively regulates pro-apoptotic ASK1 in a cell cycle-dependent way and may play a role in G2/M checkpoint-mediated apoptosis [126].

## ERK phosphorylation of CDC25C induces G2/M arrest

CDC25C is a novel MAPK ERK 1/2 target. ERK 1/2 binds and phosphorylates CDC25C on its Ser216 residue [127]. The ERK/CDC25C/CDK1/cyclin B1 pathway inhibits cell proliferation and induces G2/M arrest [128]. It has been reported that ERK1/2 regulates CDC25C activation during G2/M transition in mammalian and *X. laevis*. ERK1/2 phosphorylation of Ser216 promotes CDC25C ubiquitination and proteasomal degradation, suggesting that CDC25C proteolysis is required for sustained G2 phase arrest. The p14ARF-mediated G2 block was associated with a significant decrease in CDC25C phosphorylation at Ser216 and a significant decrease in CDC25C total protein levels. p14ARF activates MAPK ERK1/2 kinase, indicating the presence of a positive feedback loop signaling pathway linking p14ARF and MEK/ERK to control cell growth [129].

Tak1, CHK1, and CHK2, as well as proteins such as p38MAPK and MAPKAP kinase-2, are activated in normal cells in response to various external stimuli such as DNA damage and UV, thereby phosphorylating CDC25C at Ser216. Depending on the upstream signal and kinase-activated CDC25C Ser216 phosphorylation, CDC25C can also be controlled to remain in the cytoplasm or cause its degradation, contributing to its inactivation and ultimately to cell growth inhibition.

## JNK-p38MAPK negative regulation of cell cycle progression inhibits CDC25C activation

The JNK and p38MAPK pathways control cell proliferation, differentiation, survival, and migration of specific cell types, often removed in cancer. JNKs belong to the mitogen-activated protein kinase family, participate in several pathological conditions, and are thought to induce tumorigenesis to some extent by mediating stress responses or oncogenic signaling. Nonetheless, many studies have shown that JNK is involved in malignant transformation and tumor growth [130]. p38 plays the role of tumor suppressor by negatively regulating cell cycle progression and inducing apoptosis. p38 exerts carcinogenic effects such as invasion, inflammation, and angiogenesis by participating in key processes that regulate tumor progression [131, 132]. Activation of p38MAPK drives transcription, protein synthesis, cell surface receptor expression, and massive changes in cytoskeletal structure, ultimately affecting cell survival and apoptosis [133].

Recent studies have shown that JNKs may also play a role in cell cycle control. Expression of JNK is involved in regulating cell cycle progression and initiating DNA repair mechanisms. JNK directly phosphorylates CDC25C at Ser168 during the G2 phase of the cell cycle, negatively regulating its phosphatase activity, thereby modulating CDK1 activation to ensure timely control of accurate mitosis [134, 135]. CDC25C uses Ser168 as a JNK phosphate receptor site. When G2/M progression occurs (JNK is also activated), there is an enrichment of Ser168–phosphorylated CDC25C in the nucleus before CDK1 activation. These results indicate that by phosphorylation of Ser168, JNK-mediated CDC25C inhibition can prevent premature activation of phosphatase prior to mitogenesis and/or timely modulation of CDC25C activation during mitosis [136]. After DNA damage, for example under UV irradiation, induction of G2/M checkpoints requires inactivation of CDC25C by JNK phosphorylation [137]. Pectenotoxin-2 (PTX-2), an inhibitor of cytokinesis sponge, inhibits actin in leukemia, and induces increased phosphorylation of CDC25C and decreases cyclin B1/CDK1 protein level by activating the ERK-JNK pathway [138].

It has been reported that p38MAPK is involved in genistein-induced CDK1 phosphorylation and reduces CDC25C expression via the p38MAPK pathway [139]. Studies show that p38MAPK is capable of direct phosphorylation of CDC25B and CDC25C to generate a key 14-3-3 binding site for kinase activity. Under UV irradiation, p38MAPK phosphorylates CDC25B at Ser309 and 361, and CDC25C at Ser216; phosphorylation of these residues is required for binding of 14-3-3 protein; thus, p38MAPK signaling and CDC25C are related to nuclear inactivation [123].

**Fig. 1:**
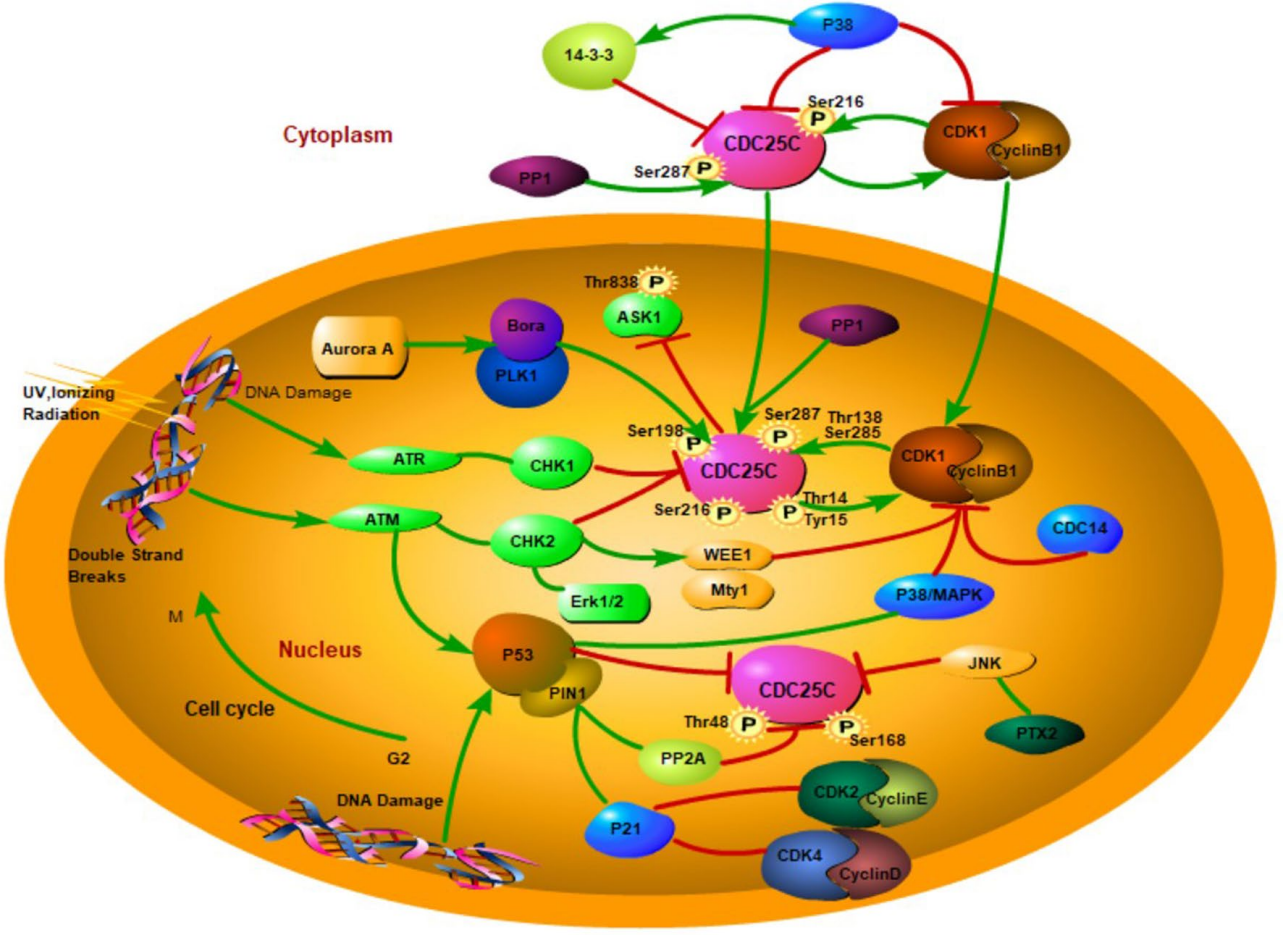
Illustration of CDC25C regulation (Green arrows indicate the enhancement effects and red indicator lines indicate the inhibitory effect). This illustration characterizes the role and activity of CDC25C in regulating the cell cycle. CDC25C and 14-3-3 protein chelate together in the cytoplasm during the interphase, and PP1 can dephosphorylate CDC25C, which can induce the releases of CDC25C in the cytoplasm in the prophase. Then CDC25C activates CDK1 to further promote the nuclear location of cyclin B1/CDK1 to initiate mitosis. In the G2/M phase, CDC25C and the cyclin B1/CDK1 complex constitutes the activation loop as the most important factor in the initiation of M phase transition and the activity of CDC25C is positively regulated by PLK1 and Aurora A. When DNA damage occurs, CHK1/CHK2, Wee1/MYT1 negatively regulates the activity of cyclin B1/CDK1 and degrades CDC25C. CDC25C is also involved in p53-mediated cell cycle arrest (other proteins including P21, P38/MAPK, PIN1/PP2A and P53 can also involve in G2/M arrest). PTX-2 can induce CDC25C phosphorylation by activating ERK-JNK pathway and decrease the level of cyclin B1-CDK1

When G2 arrest occurs, both p38MAPK and CHK1 regulatory pathways act synergistically and are required for inactivation of CDC25C and CDK1. However, their two functions do not overlap, that is, p38MAPK and CHK1 do not synergistically cause G2 arrest by mutual activation/phosphorylation, and the ATR/ATM–p38MAPK–MK2 pathway acts in parallel with the ATR–CHK1 pathway [140] (Fig. 1).

## miRNA regulates the expression of CDC25C

microRNAs (miRNA) are small endogenous non-coding RNAs with a length of about 22 nucleotides and play an important role in the differentiation, cell division, proliferation and apoptosis [141, 142]. miRNAs including miR-142-3p, miR-125b, miR-181a, miR-10b, Let-7c and miR-200b have been found to be involved in the development of diverse malignant tumors [143–145]. Other senescence-associated microRNAs can also regulate the expression of cyclins including CDC25C and cyclin B1 [146]. It is reported that miR-142-3p could directly regulate the expression of CDC25C, induce G2/M phase arrest and inhibit the proliferation of tumor cells [147–149]. miR-125b has been proved to play an anti-apoptotic role by inhibiting apoptosis regulators such as TP53 and BAK1. Overexpression of miR-125b repressed the endogenous level of P53 protein and suppressed apoptosis by regulating the expression of cyclin C and CDC25C [150, 151]. Lin et al. reported that miR-3160-5p could suppress the proliferation of prostate cancer cells by inhibiting the expression of CDC25C, CDK1 and cyclin B1 [152]. Shi et al. reported that miR-100 directly targeted the 3′ UTR region of PLK1 mRNA, inhibited the overexpression of PLK1 and further decreased the activity of CDC25C [153].

## CDC25C participates in cell apoptosis

When DNA damage occurs, G2/M checkpoint prevents mitosis to allow damage repair or initiate cell apoptosis. It is suggested that the increased expression of CDC25C may be related to anti-apoptotic signals of cancer. As the key downstream of CDC25C inhibiting apoptosis, the expression of survival protein Survivin increases in multiple malignant tumors. CDC25C could activate phosphorylation of Akt on Ser (473), increase inhibitory phosphorylation of proapoptotic BAD on Ser (136) and the expression of Survivin [154]. Survivin is also up-regulated by phosphatidylinositol-4, 5-bisphosphate 3-kinase (PI3K)-Akt signal. Overexpression of CDC25C inhibits apoptosis by stimulating PI3K/Akt signal and Survivin expression [155]. Interestingly, the key requirement for Survivin stability and anti-apoptotic function is phosphorylated by CDK1 [156].

Porcine reactive and regenerative syndrome virus (PRRSV) is an important immunosuppressive virus, which can inhibit cell proliferation and promote cell apoptosis by inactivating CDC25C, dephosphorylating CDK1 Tyr15 and inhibiting the activity of cyclin B1/CDK1 complex, and further dephosphorylating CDK1 Tyr15 [157]. Similar results were also found in Diosgenin which can induce G2/M phase arrest and promote apoptosis via activation of CHK1 kinase and CDC25C regulatory pathways [158]. Furthermore, reactive oxygen species (ROS) involves in DNA damage, cell proliferation, apoptosis and senescence by regulating the activation and expression of CDC25C [159]. It has been confirmed that ROS–p38–CDC25C–CDK1 signaling pathway mediated the phosphorylation of Survivin Thr-34 and played an important role in inducing cell cycle arrest and apoptosis [160]. CDC25C also involved in FAS-mediated apoptosis. The crosstalk between CDC25C and FAS regulated cell cycle and apoptosis [161].

## Overexpression of CDC25C and tumorigenesis

Hyperexpression of CDC25A and CDC25B in various cancers is common, but little is known about the expression of CDC25C in tumors and its mechanism of action. With intensive studies on CDC25C, researchers have gradually realized that this cell cycle regulator can play an important role in clinical treatment as a potential target for a new generation of cancer therapy. Even more surprising is that CDC25C has been shown to be a novel tumor-associated antigen. Studies have shown that in lung [4], gastric [6], bladder [7], prostate [8], esophageal squamous cell carcinoma [10], breast [162], acute myeloid leukemia [12], and colon cancers [163] CDC25C is more highly expressed than in normal tissues. According to another report, CDC25C has increased expression in female squamous cell carcinoma. The study further found that hyperphosphorylation of CDC25C Ser216 is closely related to tumorigenesis [164].

Studies in recent years have shown that the expression of CDC25C in prostate cancer is up-regulated compared to normal prostate organization, and the dephosphorylated form is nearly the only form existent. In prostate carcinoma, expression of alternative splicing variants of the CDC25C isoform are also increased, and up-regulation of expression levels is associated with the recurrence of prostate-specific antigen [9]. Similar conclusions have also appeared in liver cancer tissues [165]. The expression quantity of CDC25C was obviously increased in hepatocellular carcinoma tissues [166]; and the patients with positive expression had a longer time to relapse in primary hepatocellular carcinoma than in patients with negative expression, suggesting that CDC25C takes part in the development of liver cancer and may become an early diagnostic tool [130].

An important marker for prognosis, the protein expression level of CDC25C is an independent risk factor for postoperative mortality and survival in clinical bladder cancer. Overexpression can predict poor prognosis after radical cystectomy. CDC25C is differentially overexpressed in basal tumor cells and is associated with dedifferentiation of tumor cells. Patients with tumors with elevated CDC25C expression but not receiving chemotherapy have a higher risk of death than patients with low CDC25C expression. Interestingly, the use of chemotherapy eliminated poor prognosis associated with overexpression of CDC25C [9]. In studies of esophageal squamous cell carcinoma, overexpression of CDC25C predicts postoperative response and prognosis in patients receiving radiotherapy.

Clinical tumor biopsy specimens were analyzed by immunohistochemistry and high expression of CDC25C was detected in 27/56 (48.2%) patients. Univariate analysis showed that CDC25C overexpression and pathological complete response were associated with better survival rate [35]. In patients with FPD/AML CDC25C mutations are often found (53%). In spite of the occurrence of DNA damage, CDC25C mutations will surmount the G2/M checkpoint and facilitate cell cycle progression, suggesting that CDC25C plays a key role in the malignant transformation of FPD/AML. High expression of CDC25C was also observed in 63% of vulvar cancers, and these results further support changes in the expression of CDC25C in relation to tumorigenesis [150].

## CDC25C expression level and clinical outcome

High levels of CDC25C are advanced events in tumor development, as evidenced by the overexpression of CDC25C and the late FIGO stage, the presence or absence of lymph node metastasis, tumor size, and degree of differentiation [164]. At the same time, statistical analysis showed correlation between CDC25C overexpression and pathological complete response and survival rate. For patients receiving radiation therapy and surgery, CDC25C is independent predictor of improved survival.

Abnormal cell cycle leads to uncontrolled proliferation of cells and induces tumorigenesis. Dysfunction at the cell cycle checkpoint is considered a case marker for tumor transformation and progression. As an important cell cycle regulatory protein, CDC25C plays a vital part in regulating the initiation of mitosis, G2/M phase arrest, and response to checkpoint protein regulation. Therefore, it is reasonable to think that CDC25C can be used as a new target for cancer resistance. Overexpression of CDC25C may be related to the activation of the cyclin B1/CDK1 complex and promote the growth of the corresponding tumor, and there is a correlation between survival and proliferation rate. The available results indicate that some patients with tumors already have the immunogenicity of CDC25C, and thus CDC25C has become a new tumor marker [43]. Based on the morphological characteristics of the CDC25 family active site, it is speculated that small molecule inhibitors against CDC25C can inhibit the catalytic reaction by exposing superficial active regions, and inhibitors for CDC25A and CDC25B have been successfully developed according to structural features [167].

With the increasing progress of cancer research, radiotherapy technology has become one of the most effective ways to treat cancer. The identification of biomarkers is critical for patients who may benefit from radiation therapy. Studies have shown that G2 and M phase cells are more sensitive to radiation therapy than in other periods. CDC25C plays a significant role in the regulation of G2/M progression as an important CDK regulatory phosphatase. After knocking down the expression of CDC25C in human esophageal squamous cell carcinoma cell lines, the cells were found to decrease sensitivity to radiotherapy and inhibited cell proliferation activity. Therefore, CDC25C is considered to be a potential biomarker for radiation therapy [168]. By testing the expression of CDC25C antibody in the serum of patients with tumor, it can help to their diagnosis and treatment.

## Conclusions

As an important cell cycle regulator, CDC25 regulates G2/M progression and mediates DNA damage repair. The regulation of CDC25C in the cell cycle is affected by multiple signaling pathways and closely related to tumorigenesis and tumor development. More and more evidences showed that CDC25C could be used as a biomarker of the diagnosis and prognosis in a variety of malignant tumors. In the future, miRNA, signal inhibitors, specific antibodies, drug/peptide/small molecule based on CDC25C may be developed for the early diagnosis and clinical treatment of malignant tumors.

## Data Availability

Not applicable.

## Abbreviations

CDC25C: Cell division cycle 25C
NLS: Nuclear localization sequence
NES: Nuclear export sequence
NDS: Nuclear decision state
CRS: Cytoplasmic retention sequence
CDKs: Cyclin dependent kinases
MPF: M-phase promoting factor
ATM: Ataxia telangiectasia mutated serine/threonine kinase
ATR: Ataxia telangiectasia mutated and rad3 related kinase
CHKs: Check point kinase
Wee1: Wee1-like protein kinase
Myt1: Myelin transcription factor 1
PLK1: Serine/threonine-protein kinase 1
ARID1A: AT-rich interactive domain-containing protein 1A
Aurora A: Aurora kinase A
CUR: Curcumin
PP1: Ser/Thr protein phosphatase 1
Pin1: Peptidyl-prolyl cis–trans isomerase NIMA-interacting
PP2A: Phosphoprotein phosphatase 2A
ASK1: Apoptosis signal-regulated kinase 1
MAP3K5: Mitogen-activated protein kinase kinase kinase 5
TRX: Thioredoxin
CIP1: RNA-binding post-transcriptional regulator cip1
GADD45: Growth arrest and DNA damage inducible protein GADD45 alpha
JNK: c-Jun N-terminal kinase
PTX-2: Pectenotoxin-2
PTERK: Extracellular signal-regulated kinase
MAPK: Mitogen-activated protein kinase
Thr: Threonine
Tyr: Tyrosine
Ser: Serine
miRNA: microRNAs
3′UTRs: 3′-untranslated regions
PI3K: Phosphatidylinositol-4, 5-bisphosphate 3-kinase
ROS: Reactive oxygen species
